# CRISPR enables directed evolution in plants

**DOI:** 10.1186/s13059-019-1693-4

**Published:** 2019-04-30

**Authors:** Yingxiao Zhang, Yiping Qi

**Affiliations:** 1 Department of Plant Science and Landscape Architecture, University of Maryland, College Park, MD 20742 USA; 2 Institute for Bioscience and Biotechnology Research, University of Maryland, Rockville, MD 20850 USA

## Abstract

A proof-of-concept study has demonstrated the application of CRISPR-Cas9 for directed evolution in rice, engineering crops for desired traits.

## Introduction

**Fig. 1:**
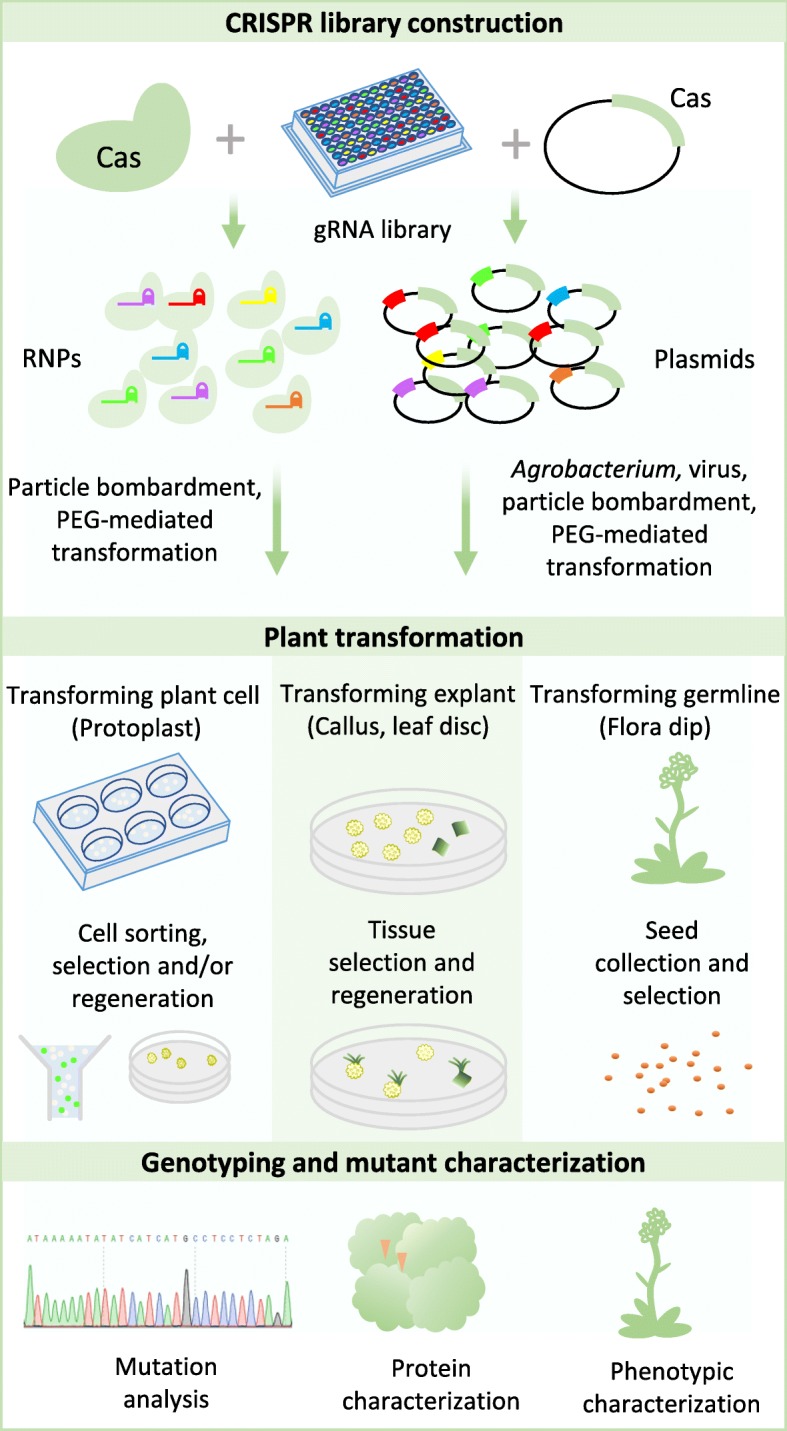
CRISPR-enabled plant directed evolution systems. Clustered regularly interspaced palindromic repeat (*CRISPR*) libraries can be constructed through ribonucleoprotein (*RNP*) assembly or cloning guide RNA (*gRNA*) library into plasmids. CRISPR libraries can be delivered into plants through *Agrobacterium*-mediated transformation, viral vectors, particle bombardment, or polyethylene glycol (*PEG*)-mediated transformation. Plant cells, explants used for regeneration, or whole plants can be used as evolution platforms. Selected mutants can be sequenced to reveal the genetic changes. Protein and phenotypic characterization can be carried out to study the evolution consequences

The clustered regularly interspaced palindromic repeats (CRISPR)-Cas9 system offers a simple, inexpensive, and efficient platform for introducing targeted mutations in both prokaryotic and eukaryotic cells. The simplicity and flexibility of multiplexed genome editing is a major advantage that the CRISPR-Cas system has over prior sequence-specific nucleases (SSNs), such as zinc finger nucleases (ZFNs) and TAL effector nucleases (TALENs). Based on RNA-guided DNA targeting, a genome-scale library of single guide RNAs (sgRNAs) can be co-delivered with Cas9 into human cells for conducting genetic screens [1]. Mutagenesis using CRISPR system targeting the whole genome or a gene family was recently demonstrated in major crops. Last year’s Chemistry Nobel prize was awarded to three pioneers of directed evolution. Directed evolution is very powerful for engineering improved or new gene functions. It elegantly combines random mutagenesis and selection and has been widely used for engineering new enzymes, antibodies, and proteins with other desired properties. On the other hand, directed evolution is conventionally and conveniently done in bacterial, yeast, or other heterologous systems. While it can be directly performed in higher eukaryotic cells such as human cells, episomal virus or DNA vector systems are typically used in such experiments [2]. However, proteins evolved in bacteria or yeast do not necessarily exhibit the same behavior in other biological systems, suggesting the importance of evolution being conducted in a native chromatin and cell environment. Plants are well suited to such a directed evolution approach, as it is now feasible to achieve targeted random mutagenesis of a plant gene of interest by coupling Cas9 with a gene-specific sgRNA library. Furthermore, whole plants can be regenerated from selected plant tissues or single cells, thanks to pluripotency, allowing for rapid phenotypic assessment of whole plants carrying newly evolved gene variants. In this issue of *Genome Biology*, Butt et al. [3] have provided a proof-of-concept study, demonstrating the use of CRISPR-Cas9 for directed evolution in rice (Fig. 1).

## A CRISPR/Cas-directed evolution platform

Butt and colleagues designed a CRISPR/Cas-directed evolution platform in which a library of all possible sgRNAs for a target gene was used. Rice *Splicing Factor 3b subunit 1* (*OsSF3B1*) was chosen as a target gene for directed evolution because it is conserved among eukaryotes and is essential for RNA splicing. In human, SF3B1 is targeted by splicing inhibitors such as herboxidiene (GEX1A) and pladienolide B (PB) [4], which have also been used as herbicides for plants, suggesting their shared mode of action in different species. After confirming the detrimental effects of genome-wide splicing repression caused by the drugs, Butt and colleagues decided to demonstrate the application of their CRISPR/Cas-directed evolution platform by evolving new variants of *OsSF3B1* that confer resistance to one of the drugs, GEX1A.

A total of 119 sgRNAs targeting the entire coding sequence of *OsSF3B1* were designed based on the NGG protospacer adjacent motif (PAM) requirement of *Streptococcus pyogenes* Cas9 (SpCas9). A total of 15,000 transformed calli were subcultured on selection medium containing GEX1A at concentrations strong enough to inhibit wild-type callus growth. Among the 21 SF3B1-GEX1A-resistant (SGR) lines regenerated from the selection medium containing 0.4 μM GEX1A, seven were further analyzed. With the protospacer sequence of each sgRNA as a barcode, it was straightforward to identify the resulting mutations at *OsSF3B1* in these lines. Most of the mutations were in-frame deletions, resulting in loss of 1 to 10 amino acids at various positions of the protein; this contrasts with the control condition without GEX1A, where no functional knockout variants were retrieved.

## Domain-focused directed evolution

Interestingly, one of the functional lines, SGR3, contains a K1050 deletion, and mutation at this amino acid position in the corresponding human homolog HsSF3B1 was previously reported to confer resistance to splicing inhibitors [5]. This encouraged the team to refine their strategy to pursue a domain-focused directed evolution, where HEAT repeats (HR) 15–17 were targeted by selected sgRNAs for mutagenesis. The same mutation carried by SGR3 was recovered again in this screen. The authors obtained three additional lines: SGR4, SGR5, and SGR6. SGR4 carried three amino acid substitutions (K1049R, K1050E, and G1051H) within *OsSF3B1*. SGR5 contained a substitution (H1048Q) and a deletion (K1049), while SGR6 had two substitutions (H1048Q and 1046S) and a deletion (K1049). All these recovered mutations are likely to affect the interaction between SF3B1 and the inhibitor GEX1A based on protein structural analysis. This domain-focused directed evolution mirrors similar practices in *Escherichia coli* or yeast, where proteins of interest can be efficiently engineered with saturation mutagenesis at chosen target domains, for instance, the engineered Cas9 variants with altered PAM requirements [6].

## Crop engineering enabled by the CRISPR/Cas-directed evolution platform

To assess germinal transmission of GEX1A resistance among SGR mutants, Butt and colleagues carried out genetic and phenotypic analysis in the next generation. Homozygous mutants were phenotypically indistinguishable from the wild-type plants, suggesting that these SF3B1 variants exhibit full splicing activity in rice. The resistance to GEX1A, however, is dose dependent and variable among SGR mutants. The mutant SGR4 displayed the strongest resistance to GEX1A. The seeds of this mutant can establish well on medium with GEX1A as high as 10 μM; under the same conditions other SGR mutants failed to germinate. Although SGR4 carried three mutations, it is likely the K1050E missense mutation has largely contributed to weakening the SF3B1 and GEX1A interaction.

This study demonstrated that it is feasible to conduct directed evolution in plants, which has significant implications. Plants evolve to adapt to their growth environments in a typically lengthy process. Accelerated evolution may provide an efficient pathway to achieving high agriculture productivity and food security in the face of global warming and climate change. Given the enormous sizes of crop genomes, it is effectively impossible to achieve saturating mutagenesis in vivo. With CRISPR, near-saturation mutagenesis becomes achievable, as shown in this study. Hence, such a directed evolution approach will be very powerful for evolving and engineering beneficial traits in crops such as herbicide resistance, improved photosynthesis, and enhanced tolerance or resistance to abiotic or biotic stresses. Genomics tools such as genome-wide association studies (GWASs) have aided rapid mapping and discovery of important genes and alleles in crop productivity and stress tolerance in major crops. It is anticipated that CRISPR-enabled directed evolution on these target genes would help improve agronomic traits or generate novel alleles directly in elite cultivars. While directed evolution is often used for protein engineering, it is also possible to apply it towards engineering quantitative traits by targeting *cis-*regulatory elements [7]. Furthermore, we envision that similar directed evolution approaches can be practiced in animals as well, such as worm, fruit fly, and zebrafish.

## Concluding remarks

While CRISPR-Cas9 was used in this study, directed evolution in plants could be further empowered by other CRISPR systems. The majority of CRISPR-Cas9-induced mutations in rice are insertions and deletions (Indels), which are more likely to generate frame-shift mutations than in-frame mutations. This is especially an issue when knockout mutants are not viable or heritable, making mutagenesis power wasted during selection. As shown in this study, half of the six SGR mutants carried missense mutations, suggesting the importance of this mutation type in evolving new protein function. Moreover, the most common type of variations in nature are single nucleotide polymorphisms (SNPs), demonstrating the need to generate substitutions instead of indels, especially for gain-of-function mutations. The development of CRISPR-derived hypermutators, including CRISPR-X [8], targeted AID-mediated mutagenesis (TAM) [9] and EvolvR [10], enables targeted diversifying of genes and genomic regions. By using different deaminases and recruiting systems, target window and nucleotide changes can be tuned accordingly, potentially making mutagenesis not limited by PAM.

To improve the evolving force, the targeting scale can be enlarged through multiple strategies (Fig. 1). CRISPR with altered PAM requirements may be used, such as orthogonal Cas9 proteins, engineered SpCas9 variants, and other CRISPR systems (e.g., Cas12a and Cas12b). There is also potential to optimize the delivery methods to maximize sgRNAs transformed into plants. Moreover, other platforms, such as plant cells, could be used for directed evolution. In sum, the door is wide open for in vivo directed evolution in eukaryotes, which will be further enhanced with the rapid development of CRISPR genome editing technologies down the road.

## Abbreviations

CRISPR: Clustered regularly interspaced palindromic repeat
PAM: Protospacer adjacent motif
SGR: SF3B1-GEX1A-resistant
sgRNA: Single guide RNA
